# Glucose lowering effect of transgenic human insulin-like growth factor-I from rice: *in vitro *and *in vivo *studies

**DOI:** 10.1186/1472-6750-11-37

**Published:** 2011-04-12

**Authors:** Stanley CK Cheung, Li-zhong Liu, Lin-lin Lan, Qiao-quan Liu, Samuel SM Sun, Juliana CN Chan, Peter CY Tong

**Affiliations:** 1 Department of Medicine and Therapeutics, The Chinese University of Hong Kong, Prince of Wales Hospital, Shatin, Hong Kong; 2 Department of Biology, The Chinese University of Hong Kong, Shatin, Hong Kong; 3 Key Laboratory of Plant Functional Genomics of the Ministry of Education, Agricultural College, Yangzhou University, Jiangsu 225009, PR China

**Keywords:** Oryza sativa L., plant bioreactor, transgenic plant, recombinant protein, protein targeting, KDEL, IGF-I

## Abstract

**Background:**

Human insulin-like growth factor-I (hIGF-I) is a growth factor which is highly resemble to insulin. It is essential for cell proliferation and has been proposed for treatment of various endocrine-associated diseases including growth hormone insensitivity syndrome and diabetes mellitus. In the present study, an efficient plant expression system was developed to produce biologically active recombinant hIGF-I (rhIGF-I) in transgenic rice grains.

**Results:**

The plant-codon-optimized hIGF-I was introduced into rice via *Agrobacterium*-mediated transformation. To enhance the stability and yield of rhIGF-I, the endoplasmic reticulum-retention signal and glutelin signal peptide were used to deliver rhIGF-I to endoplasmic reticulum for stable accumulation. We found that only glutelin signal peptide could lead to successful expression of hIGF-I  and one gram of hIGF-I  rice grain possessed the maximum activity level equivalent to 3.2 micro molar of commercial rhIGF-I. *In vitro *functional analysis showed that the rice-derived rhIGF-I was effective in inducing membrane ruffling and glucose uptake on rat skeletal muscle cells. Oral meal test with rice-containing rhIGF-I acutely reduced blood glucose levels in streptozotocin-induced and Zucker diabetic rats, whereas it had no effect in normal rats.

**Conclusion:**

Our findings provided an alternative expression system to produce large quantities of biologically active rhIGF-I. The provision of large quantity of recombinant proteins will promote further research on the therapeutic potential of rhIGF-I.

## Background

Human insulin-like growth factor-I (hIGF-I) plays a critical role in cellular differentiation, proliferation, growth and apoptosis [1]. It is a single polypeptide chain of 70 amino acid residues and is encoded by a single gene on chromosome 12, with a molecular weight of 7646 Da [2,3]. It has 50% amino acid sequence homology with insulin. Hence, hIGF-I has been proposed as an alternative therapeutic agent to treat diabetic mellitus, especially for those patients with defects in insulin receptors. Apart from diabetes mellitus, the efficacy of hIGF-I in the treatment of growth disorders as well as insulin resistance has been studied [4]. In late 2005, recombinant hIGF-I (rhIGF-I) was approved by the United States Food and Drug Administration (USFDA) as a therapeutic agent for the growth hormone insensitivity syndrome (GHIS) [5].

Recombinant hIGF-I was first synthesized by recombinant DNA techniques in 1986. To date, commercial rhIGF-I is mainly produced in different organisms [6-8]. Problems of these processes include low expression levels, high equipment and production costs, incorrect post-translational modifications as well as potential contamination with human pathogens. With the advance in genetic engineering, rice is recently recognized as a promising alternative for the production of safe and economical biopharmaceutical in large quantities [9]. Rice offers the advantage of producing large quantity of proteins in terms of cost, product safety, scalability and authenticity [9]. Rice is known to exclude any noxious chemicals such as nicotine and toxic alkaloids in tobacco as well as having low allergenicity. Large amount of recombinant proteins can be synthesized at one time as a single rice plant can produce over 1,000 grains. Besides, as rice is self-pollinated, simple regulatory rules like isolation distances can be set up to prevent cross-pollinating and out crossing of the transgenic trait.

In an attempt to achieve high-yield expression of foreign genes in plants, the coding sequence of heterologous gene has to be modified to plant-preferred codons. Previous studies have showed that codon usage biases are strongly correlated with gene expression levels [10]. Highly expressed genes preferentially use a subset of "optimal" codons which correspond to the most abundant tRNAs, leading to enhanced translation accuracy and efficiency [11,12]. Moreover, proteins yields can be increased if the protein is directed to specific compartments in order to prevent degradation by the proteolytic system of the cells. In a plant cell (as in all eukaryotic cells), an amino-terminal signal peptide can direct proteins to the secretory pathway, including the endoplasmic reticulum (ER), the Golgi complex and hydrolytic compartments (vacuoles in plants), or to secretion from the cell. It has been found that secretory proteins could be accumulated to high level than those expressed in cytosol [13]. Some studies found that proteins yields can be further enhanced if the protein is retained in the ER lumen using the Lys-Asp-Glu-Leu (KDEL) C-terminal tetrapeptide [14]. Protein levels were 6-14 times higher in cells transformed with the construct containing KDEL than that without KDEL [15].

In the present study, we hypothesized that functional rhIGF-I could be produced in transgenic rice grains. Codons of cDNA of hIGF-I was modified to plant-preferred sequences [16] and introduced into rice by *Agrobacterium*-mediated transformation. Glutelin signal peptide and KDEL tetrapeptide were included for expression enhancement of recombinant proteins. Biological activity of rhIGF-I was confirmed by the induction of membrane ruffles and the increase in glucose transport in rat skeletal muscle cells (L6myc). *In vivo *study demonstrated that transgenic rhIGF-I from rice reduced blood glucose level in streptozotocin induced diabetic and Zucker fatty rats. These findings confirmed that biologically active rhIGF-I could be produced in the rice-based system.

## Results

### Expression of rhIGF-I in transgenic rice seeds

**Figure 1:**
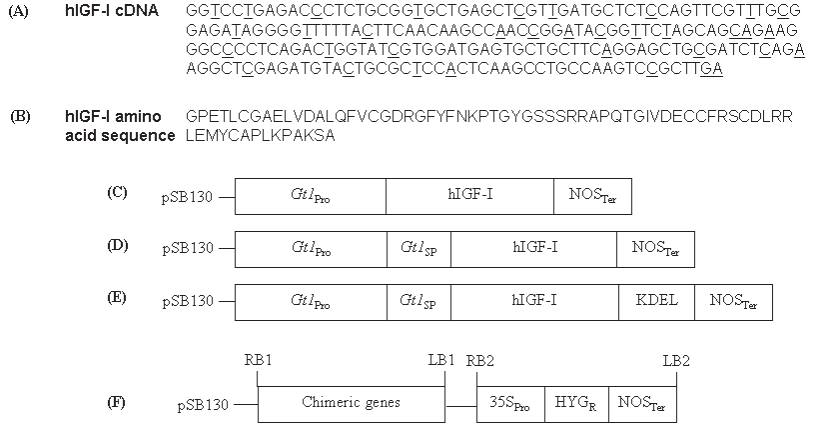
**Expression of hIGF-I in transgenic rice**. (A) Nucleotide sequence of the modified hIGF-I cDNA. The modified nucleotide is underlined and the change of codon was 28.6%. (B) The hIGF-I protein is predicted to contain 70 amino acids. (C - E) The recombinant hIGF-I expression constructs, driven by glutelin promoter, used for rice transformation. (C) The construct, pSB130/Gt1/hIGF-I (I) contained the modified hIGF-I alone. (D) The construct, pSB130/*Gt1*/SP/hIGF-I (SI) contained glutelin signal peptide only (E) whereas the pSB130/*Gt1*/SP/hIGF-I/KEDL (SIK) construct carried both the glutelin signal peptide and KDEL. (F) All the chimeric genes were ligated into the twin T-DNA binary vector, pSB130, for *Agrobacterium *transformation. The pSB130 vector, contains two T-DNAs, one flanking the gene of interest driven by Gt1 promoter while the other flanking the selectable marker, hygromycin phosphotransferase (HYG). (Abbreviations: RB - right border; LB - left border.)

**Figure 2:**
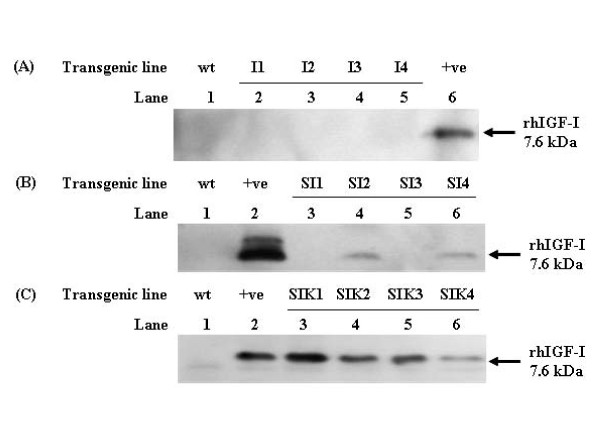
**Western blot analysis of different transgenic rice seeds**. Total protein (150 ug) was extracted from mature rice seeds of different transformants, blotted onto PVDF membrane. Recombinant rhIGF-I protein was used as positive controls. (A) Lane 1: wild type rice plant (wt); lanes 2-5: pSB/*Gt1*/hIGF-I transformants I1 to I4; lane 6: positive control (+ve) - commercial rhIGF-I protein. (B) Lane 1: wild type rice plant (wt); lane 2: positive control (+ve) - commercial rhIGF-I protein; lanes 3-6: pSB/*Gt1*/SP/hIGF-I transformants SI1 to SI4. (C) Lane 1: wild type rice plant (wt); lane 2: positive control (+ve) - commercial rhIGF-I protein; lanes 3-6: pSB/*Gt1*/SP/hIGF-I::KDEL transformants SIK1 to SIK4.

To increase the expression level of hIGF-I, glutelin signal peptide (SP) and ER-retention signal KDEL were added to the chimeric constructs. Three chimeric gene constructs (I, SI and SIK) were designed and transferred into rice by *Agrobacterium*-mediated transformation (Figure 1). Southern blot analysis confirmed the integration of constructs (I, SI and SIK) into the rice genome (Additional file 1 Figure S1). As the restriction enzyme BamHI cuts only once in all expression constructs, the number of bands represented the number of copies of the transgenes introduced into rice. Most of the transformants contained 1 to 2 copies of the transgenes, while no signal was detected in wild type plant. Total seed protein extracts from different transformants were examined by Tricine SDS-PAGE and Western blot. A distinct band of about 7.6 kDa was observed in transformants SI and SIK (Figure 2B and 2C), whereas no signals were detected in transformant I and wild type rice plant (Figure 2A). These results suggested that glutelin SP was essential for the expression of the transgenes.

### Ruffling effect of commercial and rice-produced rhIGF-I

**Figure 3:**
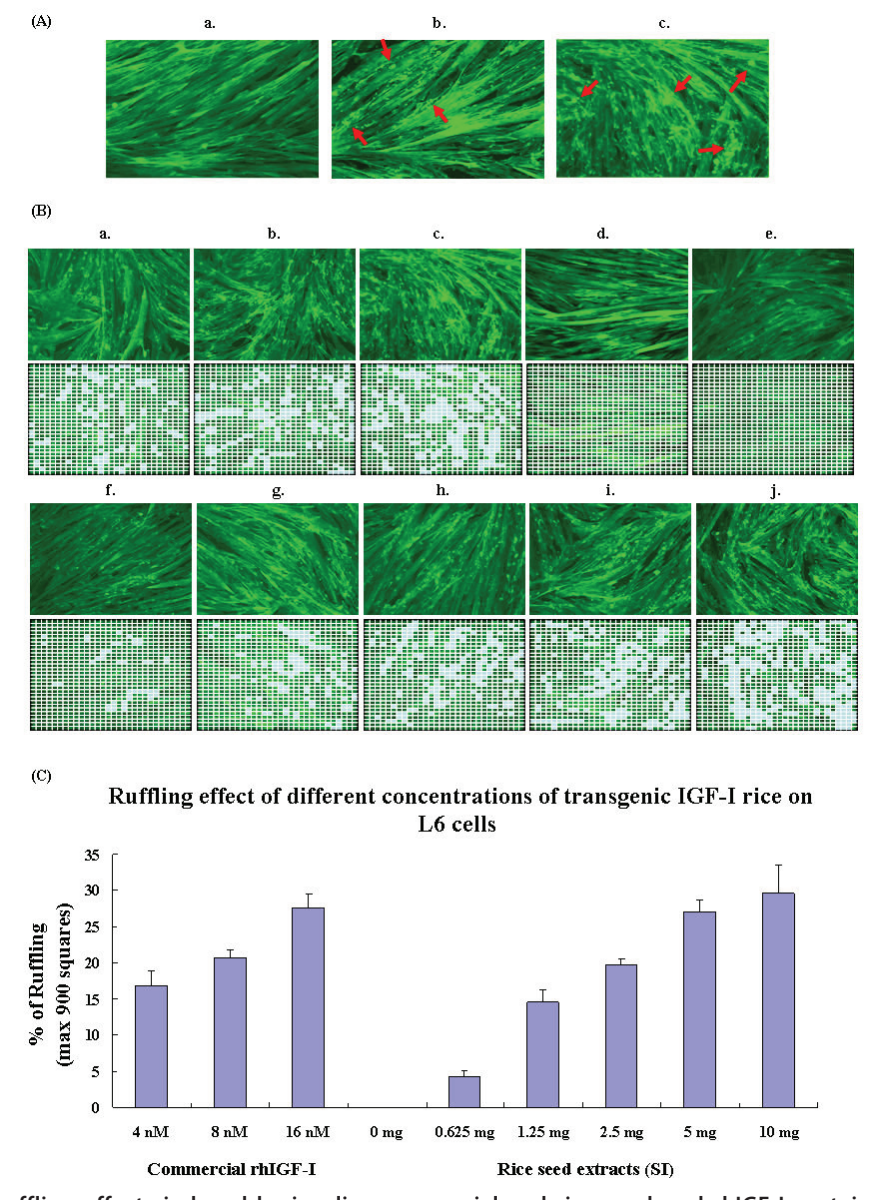
**Membrane ruffling effects induced by insulin, commercial and rice-produced rhIGF-I proteins on L6 myotubes**. (A) Morphology of membrane ruffling effects induced by insulin and commercial rhIGF-I (200×). *a*) Normal L6 muscle cells. After adding *b*) 100 nM of insulin and *c*) 16 nM of commercial rhIGF-I, the actin filaments of L6 cells were reorganized and looked like human winkle. (B) Morphology of membrane ruffling effects induced by commercial and rice-produced rhIGF-I (SI) (200×). The ruffling effect was estimated by dividing each results photo into nine hundreds equal squares. Commercial rhIGF-I (4, 8 and 16 nM), SI seed crude protein extract (0.625 - 10 mg) were used to treat L6 cells. Wild type rice (12 mg) seed crude protein and protein extraction buffer (basal) were the negative controls. The average number of squares showing ruffling in any three photos was counted and the percentage of ruffling was calculated. The squares showing ruffling were marked in blue. *a*) Commercial rhIGF-I 4 nM; *b*) Commercial rhIGF-I 8 nM; *c*) Commercial rhIGF-I 16 nM; *d*) Wild type rice 12 mg; *e*) Protein extraction buffer, basal; *f*) SI 0.625 mg; *g*) SI 1.25 mg; *h*) SI 2.5 mg; *i*) SI 5 mg; J) SI 10 mg. (C) A graph showing membrane ruffling effects induced by commercial and rice-produced rhIGF-I (SI). The three columns on the left hand side represented the ruffling effect induced by 4, 8 and 16 nM of commercial rhIGF-I while the remaining indicated membrane ruffling caused by 0 - 10 mg of SI seed crude protein. Data are shown as means ± SD.

Growth factors such as IGF-I and insulin are known to cause reorganization of actin filament leading to membrane ruffles [17]. Using the rat skeletal muscle cells (L6myc), we compared the effect on actin remodeling of rhIGF-I from commercial source and transgenic rice. We found that the morphological change induced by 16 nM of commercial rhIGF-I was equivalent to that of 100 nM of insulin (Figure 3A). To study the *in vitro *function of rice-produced rhIGF-I, seed crude protein extracts of various amount from different transformants (0, 0.625, 1.25, 2.5, 5 and 10 mg) and commercial rhIGF-I (4, 8 and 16 nM) were added to L6myc cells. Membrane ruffles were found only when SI protein was added (Figure 3B), while no morphological changes were observed when L6myc cells were treated with I, SIK (data not shown) or wild type rice (Figure 3B d). As depicted in Figure 3C, the ruffling effect induced by SI protein was directly proportional to the amount of the protein added. Extract containing 5 mg of SI protein caused membrane ruffling similar to that of 16 nM of commercial rhIGF-I (area of ruffles: 27.00 ± 1.73% vs. 27.60 ± 1.92%).

### Glucose uptake assay

**Figure 4:**
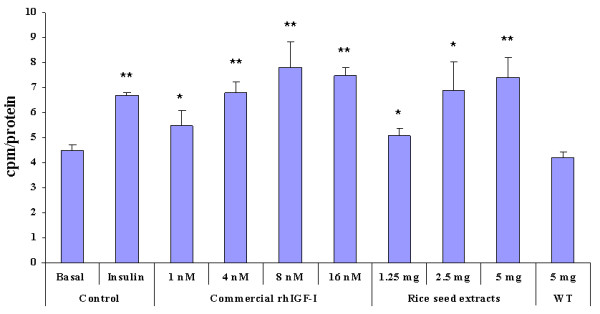
**Comparison of glucose uptake effects between commercial rhIGF-I and transgenic rice seed extracts (SI) on L6 cells**. Different concentrations of commercial rhIGF-I (1 nM to 16 nM) and SI seed crude proteins (1.25 mg to 5 mg) were added to the L6 cells and the effects on glucose uptake were measured. Insulin (100 nM) was the positive control while basal was the negative control. Data are shown as means ± SD. *p < 0.05, **p < 0.01.

The metabolic effect of rhIGF-I was studied by measuring the glucose uptake in L6myc cells. Only SI protein was tested as it possessed biological activity as demonstrated by membrane ruffle experiments. Different concentrations of commercial rhIGF-I (1, 4, 8 and 16 nM) and SI protein (1.25, 2.5 and 5 mg) were added to L6myc cells. Insulin (100 nM) was used as positive control. As depicted in Figure 4, both commercial rhIGF-I and SI proteins stimulated glucose uptake in a dose-dependent manner. Five mg of SI protein was found to cause comparable glucose uptake as 8 nM of commercial rhIGF-I (7.40 ± 0.79 cpm/protein vs. 7.80 ± 1.03 cpm/protein). No significant difference in glucose uptake was observed when basal or wild type rice seed proteins were added.

### In vivo feeding test

**Figure 5:**
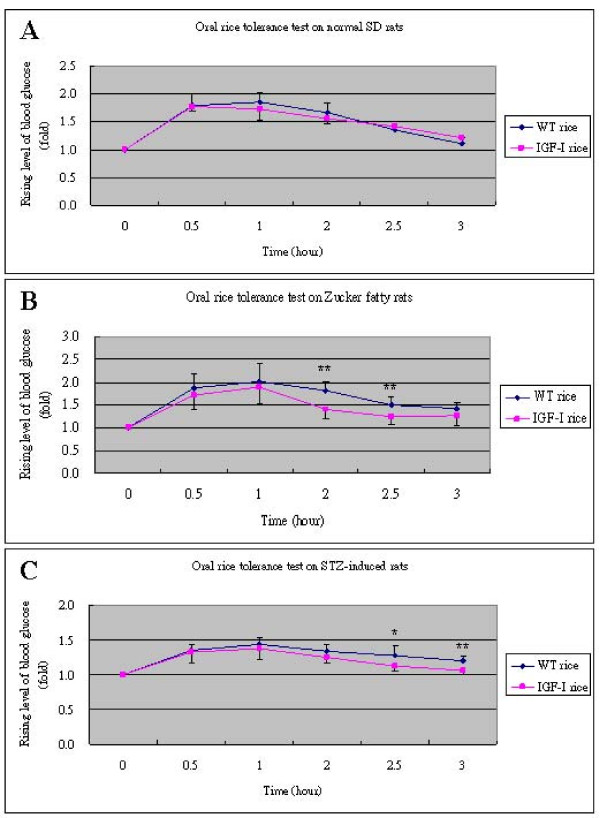
**Comparisons of oral rice tolerance test (ORTT) between rice seed extracts of wild type rice (WT) and transgenic IGF-I rice (SI) on different type of rats**. (A) Normal SD rats (n = 5), (B) Zucker fatty rats (n = 8) and (C) STZ rats (n = 8). Blood glucose levels at different time points were measured by multiple of increase after feeding with rice extract. Data are shown as means ± SD. *p < 0.05, **p < 0.01.

To determine *in vivo *glucose lowering effect, different animal models of diabetes, namely streptozotocin-induced diabetic rats (STZ, a model of type 1 diabetes, n = 8) and Zucker diabetic fatty rats (ZDF, a model of type 2 diabetes, n = 8) were fed with transgenic rhIGF-I rice (SI). Sprague-Dawley (SD) rats were used as control (n = 5). Plasma glucose levels of SD rats fed with SI rice were similar to those fed with wild type rice (Figure 5A). In Zucker fatty rats (Figure 5B) and STZ-induced diabetic rats (Figure 5C), ingestion of SI rice was associated with lower plasma glucose levels when compared with those fed with wild type rice. Significant differences were observed at 2 hours (1.41 ± 0.20 fold vs. 1.82 ± 0.19 fold, *p *= 0.001) and 2.5 hours (1.25 ± 0.18 fold vs. 1.51 ± 0.17 fold, *p *= 0.009) for Zucker fatty rats, while for STZ-induced diabetic rats, significant differences occurred at 2.5 hours (1.12 ± 0.07 fold vs. 1.28 ± 0.14 fold, *p *= 0.01) and 3 hours (1.06 ± 0.06 fold vs. 1.19 ± 0.07 fold, *p *= 0.001). The results indicated that SI rice reduced blood glucose in Zucker fatty and STZ-induced diabetic rats, though it had no effect on blood glucose level in normal SD rats.

### Starch contents of rice grains

Total starch analysis was performed to confirm the endogenous starch contents of SI transgenic rice and wild type rice. The OD reading of SI and wild type rice were 0.574 ± 0.019 vs. 0.590 ± 0.023, *p *= 0.119) respectively (Additional file 2 Figure S2). The result suggested that both transgenic and wild type rice grains had similar starch content.

## Discussion

This is the first report to demonstrate the expression of native rhIGF-I in transgenic rice grains. Various strategies were employed in the design of the chimeric constructs to enhance the stable expression of the human recombinant proteins in plant. Firstly, the codons of human IGF-I gene were modified to plant-preferred sequence. Secondly, sequences of two plant seed storage proteins, lysine-rich protein (LRP) from winged bean and methionine-rich 2S albumin from Paradise nut [16] were included in constructs for high protein expression and stable accumulation [18,19]. Thirdly, as subcellular targeting is another crucial factor in determining the expression of foreign proteins, rice glutelin signal peptide (SP) and ER-retention sequence KDEL were employed for stable accumulation of rhIGF-I in transgenic rice grains. Western blot analysis showed that only constructs containing SP and KDEL (SI and SIK) were expressed in transgenic rice grains (Figure 2). We postulated that the addition of SP promotes the inclusion of candidate proteins SI and SIK into the secretory pathway of the endoplasmic reticulum (ER) [20]. After the cleavage of SP in the ER, the SI protein, in the absence of further protein sorting sequences, went through the Golgi apparatus and was finally secreted out of the cell in the intercellular space for storage. In contrast, the SIK protein was re-directed and retained in the ER by the presence of KDEL sequence. The KDEL tetrapeptide contributes to protein localization by interacting with a receptor that recycles between the Golgi apparatus and the ER [14]. The oxidizing environment and the presence of molecular chaperones in ER promote protein assembly and folding. Furthermore, few proteases are present in ER for degradation of proteins. Hence, both SI and SIK proteins were not degraded by protease and could be stably accumulated. Our results were consistent with the increased accumulation of vicilin, a pea vacuole storage protein in transgenic alfalfa leaves when the KDEL signal was included in C-terminus [21].

Despite being expressed in the transgenic rice grain, SIK protein failed to induce membrane ruffling in L6myc cells. This results suggested that SIK protein was devoid of biological activity of IGF-I. In native human IGF-I protein, there are no glycosylation site. A possible explanation of the difference in biological activity between SI and SIK proteins could be related to the glycosylation of candidate protein. The presence of the KDEL sequence has been shown to affect the extent of glycosylation in the ER and Golgi apparatus [22]. It is feasible that the presence of the KDEL sequence in SIK proteins might enhance the glycosylation of candidate proteins. Further study on the presence of glycosylation in SIK will be required to confirm the hypothesis. Other possible explanation includes the misfolding of candidate protein while in the ER. The increase in SIK protein in the ER may lead to ER stress, resulting in over-expression of ER chaperones, luminal binding protein (BiP) and the protein folding catalyst, protein disulphide-isomerase [23]. Previous studies had reported that over-expression of BiP had inhibitory effect on protein translation, folding and deposition of storage proteins into rice endosperm [23,24]. Our results were consistent with previous study in which the fluorescence intensity of green fluorescent protein with KDEL was reduced as a result of protein misfolding [25].

The membrane ruffling and glucose uptake in rat skeletal muscle cells (L6myc) were used to evaluate the biological activity of transgenic rhIGF-I. Growth factors including IGF-I are known to cause rapid actin remodeling leading to membrane ruffles [17]. The IGF-I has been shown to be about 10 - 100 times more potent than insulin in causing membrane ruffles [26]. In the present study, 16 nM of commercial rhIGF-I was found to have similar effect in membrane ruffles as compared to 100 nM of insulin in L6myc. Importantly, rice-produced rhIGF-I (SI) was found to induce similar membrane ruffling as commercial rhIGF-I and the effect was dose-dependent (Figures 3B and 3C). In term of membrane ruffles, one gram of SI rice grain possessed equivalent activity to 3.2 μM of commercial rhIGF-I. For glucose uptake study, 5 mg of SI protein was found to be equivalent to 8 nM of commercial rhIGF-I (Figure 4). Hence, it was estimated that one gram of SI rice grain contained 1.6 μM of functional rhIGF-I. Taken together, these results indicated that the properties of rice-produced rhIGF-I were quite similar to those of the native IGF-I and insulin on membrane ruffles and glucose uptake.

Schoenle *et al*. previously showed that treatment with IGF-I reduced both insulin and blood glucose levels in patients with severe insulin resistance [27]. It has been postulated that IGF-I might enhance insulin secretion and sensitivity [28,29], stimulate β cell regeneration [30] as well as prevent β cell from apoptosis [31,32] in patients with diabetes. To evaluate whether the rice-derived rhIGF-I could also lower blood glucose level, an oral rice tolerance test (ORTT) was developed based upon the methodology of oral glucose tolerance test. As starch contributes 80% of rice grain, glucose released after digestion will lead to higher blood glucose level, which is similar to the effect of glucose intake. We found that SI could efficiently lower blood glucose levels in Zucker and STZ-induced rats (Figures 5B and 5C), whereas it did not have significant effect in normal SD rats (Figure 5A) when compared with wild type rice. Blood glucose levels only showed significant differences after 2 hours in Zucker fatty rats and 2.5 hours in STZ rats. This time delay may be due to the release of rice-derived rhIGF-I after digestion of SI rice grain powder. When feeding the three types of rats with equal amount of commercial rhIGF-I, no significant differences were found in blood glucose levels (data not shown), suggesting that commercial rhIGF-I might be totally degraded as it went through the gastrointestinal tract in rats. On the other hand, rice grain might help protect the rice-derived rhIGF-I from the digestive system in rats. This effect might be attributed to the location of rhIGF-I accumulated in rice grain. Seed protein is normally deposited in storage organelles known as protein bodies [33]. Studies found that protein bodies can withstand the harsh acidic environment of the digestive tract in rats, making it possible for inducing antigen-specific immune responses by oral administration of rice-based mucosal vaccine [34].

To scrutinize whether the blood glucose lowering effect was solely resulted from the action of the rhIGF-I in SI rice, total starch contnet of SI and wild type rice grains was assessed by enzymatic digestion with α-amylase and amyloglucosidase (Megazyme). As depicted in Supplemental Figure S2, there was no significant difference in endogenous starch contents between SI and wild type rice grains. Our results confirmed that the blood glucose lowering effect was solely due to the action of rice-derived rhIGF-I.

Recent study demonstrated that rice-codon-modified rhIGF-1 could also be expressed in transgenic rice grains. Oral administration of the codon-modified rhIGF-I could reduce blood glucose in diabetic mice by enhancing plasma rhIGF-I level. The rhIGF-I produced by Xie *et al*. was a fusion protein of 38 kDa, with a C-terminal ER luminal binding protein, but not native rhIGF-I [35]. In the present study, native rhIGF-I with about 7.6 kDa (Figure 2B) could successfully be expressed and its biological functions were similar to those of commercial rhIGF-I, as indicated in both *in vitro *(Figures 3, 4) and *in vivo *(Figure 5) analysis. Hence, our approach might provide an alternative methodology in producing native rhIGF-I. Using a different approach, rhIGF-I was expressed in rice leaves [36]. Further extraction and purification of the protein from leaves would be required before the protein could be utilized. The expression of rhIGF-I in rice grain in the present study represents a better way of producing functional rhIGF-I that possesses insulin-like biological functions for clinical applications.

The limitation of this report is that it is only a preliminary study to test the feasibility of hIGF-I expression in transgenic rice. The *in vivo *study we performed was an acute effect of the rice-derived rhIGF-I on rats. Long-term study will be carried out to see whether the rhIGF-I possesses glucose lowering effect and its side effects in diabetic rats. Besides, the level of IGF-I will be measured to see whether the glucose lowering effect is attributed to the rice-derived rhIGF-I. Moreover, as the rice-derived rhIGF-I was neither isolated nor purified, attempts will be put on purification of the rice-derived rhIGF-I for possible clinical uses.

## Conclusion

We have successfully expressed native rhIGF-I in rice grains using transgenic approach. Plant-optimized hIGF-I cDNA was incorporated in different expression constructs and introduced into rice by *Agrobacterium*-mediated transformation. One gram of SI rice grain possessed the maximum activity level equivalent to 3.2 μM of commercial rhIGF-I. *In vitro *studies showed that rice-derived rhIGF-I could induce membrane ruffling and enhance glucose uptake in L6myc cells. Oral administration of transgenic rhIGF-I rice confirmed that it reduced plasma glucose levels in STZ-induced and Zucker diabetic fatty rats, while it did not have significant effect in normal SD rats. Taken together, our findings suggested that biologically active rhIGF-I could be produced in transgenic rice.

## Methods

### Chimeric genes construction

The hIGF-I cDNA [37] was first modified to enhance its expression in plant, with 28.6% change of codons (Figure 1A) [16]. Three constructs were designed, namely promoter only construct (I), signal peptide construct (SI) and protein sorting construct (SIK). All of them were cloned into the twin T-DNA binary vector, pSB130, for *Agrobacterium*-mediated transformation in rice [38]. The binary vector contains two T-DNAs, one flanking the gene of interest driven by rice glutelin *Gt1 *promoter while the other flanking the selectable marker, hygromycin phosphotransferase (HYG) (Figure 1F). It is believed that the two independent T-DNAs will be integrated into different chromosomes or the same chromosome in different locations. By independent segregation of the chromosomes, selectable marker-free transgenic rice with the transgene only can be produced. For the promoter only construct, the plant codon-optimized hIGF-I cDNA was first amplified to introduce a 5' BamHI and 3' KpnI restriction sites by PCR using primer IBL, 5' - CGGGATCCATGGGTCCTGAGACCCTC - 3' and IKR, 5'-GGACGGTTCAGGCGAACTCCATGGGG - 3'. The PCR product was ligated into the vector pSB130, resulting in pSB130/*Gt1*/hIGF-I (I) (Figure 1C). The signal peptide construct was made same as the procedure described above except for the primers used. Primers INL, 5'-CATGCCATGGGTCCTGAGACCCTCTGC - 3' and IKR were used to introduce NcoI and KpnI sites to the gene fragment. The resulting construct, pSB130/*Gt1*/SP/hIGF-I (SI), contained the modified hIGF-I and glutelin signal peptide (Figure 1D). For the protein sorting construct, a 5' NcoI restriction site and a 3' KDEL protein sorting sequence were added to the target gene by using the primers INL and IKKR, 5' - GAGTTCGGACGGTTCAGGCGATTTCTACTCGATACTCCATGGGG - 3'. The final construct was pSB130/*Gt1*/SP/hIGF-I/KDEL (SIK) (Figure 1E). To confirm the sequence fidelity of the chimeric genes, cycle sequencing was carried out using ABI PRISM^® ^dRhodamine Terminator cycle sequencing kit (Applied Biosystems) and the results were analyzed using ABI PRISM^® ^3100 Genetic Analyzer (Applied Biosystems) as described in the user manual. The final constructs with correct sequences were transferred into *Agrobacterium tumefaciens *strain *EHA105 *by heat-shock method [38] for rice transformation.

### Rice transformation and selection

Mature rice seeds of an elite *japonica *rice variety Wuyunjing 9 from China were used for *Agrobacterium*-mediated transformation previously described [38]. Briefly, mature rice seeds were first sterilized with 50% Clorox (5.25% sodium hypochlorite) together with 1 drop of Tween 20. Sterilized rice seeds were cultured in callus induction medium for callus formation. Newly-formed calli were excised from the endosperm and immerged in *Agrobacterium *culture harbouring the expression constructs. Infected calli were placed in selection medium to allow resistant calli to form. Resistant calli were removed from the original sample and cultured in pre-regeneration and regeneration medium for shoots formation. Roots were allowed to grow in rooting medium and finally the plantlets were transferred to soil and grown in greenhouse.

### Southern blot analysis

Leaf genomic DNA of T0 transgenic rice plant was isolated by the cetyltrimethylammonium bromide (CTAB) method as described previously [39]. Fifteen μg of genomic DNA was digested overnight with BamHI, separated on 0.8% agarose gel and transferred to positively charged nylon membrane (Roche) using the VacuGeneXL Vacuum blotting System (Pharmacia Biotech). Hybridization and detection were performed in accordance with the method described in the DIG Nucleic Acid Detection Kit (Roche). Human IGF-I specific bands were detected by denatured DIG-labeled hIGF-I probe prepared by PCR using DIG DNA labeling Kit (Roche).

### Western blot analysis

Total seed protein was obtained from grinding mature T1 transgenic rice seeds (20 mg) into powder and mixed with 80 μl of Tris extraction buffer (0.125 M Tris-HCl, 0.1% SDS, pH 6.8). The quantity of seed protein was determined by the bicinochoninic acid (BCA) method and bovine serum albumin (BSA) was used as standard. For Western blot analysis, 150 μg each of total seed protein extracts were resolved in 10% Tricine SDS-PAGE and blotted on PVDF membrane (Bio-Rad) using Towbin buffer (48 mM Tris, 39 mM Glycine and 20% methanol). The membrane was then incubated in primary anti-IGF-I (Santa Cruz Biotechnology, Inc) polyclonal antibody, followed by AP-conjugated anti-rabbit immuno-globulin G (Bio-Rad) at a 1:30000 dilution. Finally the membrane was subjected to non-radioactive detection with chemiluminescent Starlight™ Substrate (ICN) as described in the manual of Aurora™ Western Blot Chemiluminescent Detection System (ICN).

### Functional analysis of rice-produced rhIGF-I

#### A) Membrane ruffling

Rat L6 skeletal muscle cells expressing c-myc epitope-tagged glucose transporter 4 (GLUT4) (L6myc cells) [40,41] were maintained in myoblast monolayer culture in α-minimal essential medium containing 10% (v/v) fetal bovine serum (FBS) and 1% (v/v) antibiotic-antimycotic solution (100 U/ml penicillin G, 10 mg/ml streptomycin and 25 mg/ml amphotericin B) in an atmosphere of 5% CO_2 _at 37°C. Cells were subcultured by trypsinization of subconfluent cultures using 0.25% trypsin. Myoblasts were plated in medium containing 2% (v/v) FBS at approximately 4 × 10^4 ^cells/ml for 5-7 days for differentiation into myotubes. The differentiated myotubes were deprived of serum for 3 hours and treated with different concentrations of rhIGF-I extracted from transgenic rice seeds for 10 minutes at 37°C. After incubation, the myotubes were fixed with 3% (v/v) ice-cold paraformaldehyde and permeabilized with 0.1% (v/v) Triton X-100. After blocking in 0.1% BSA, the myotubes were incubated in Phaloidin and then mounted in ProLong Antifade solution onto glass slides. Samples were examined with a Zeiss Axioplan 2 imaging microscope and a Zeiss LSM 510 META laser scanning confocal microscope (Carl Zeiss, Jena, Germany). Confocal image was divided into 900 equal squares for determination of ruffling intensity. Three images were collected in each group and the number of squares showing ruffling was counted, taken average and calculated.

#### B) 2-Deoxy-[3H]deoxyglucose Uptake

After 24 hours starvation, L6myc myotubes were left untreated or treated with 100 nmol/l insulin or different concentrations of transgenic rice seed protein for specific period at 37°C. Subsequently, cells were washed several times with glucose-free HEPES-buffered saline solution (140 mmol/l NaCl, 20 mmol/l Na-HEPES, pH 7.4, 2.5 mmol/l MgSO_4_, 5 mmol/l KCl, and 1 mmol/l, CaCl_2_). Glucose uptake was measured as described previously by using 2-deoxy-[3^H^] deoxy-glucose [42] and each condition was assayed in triplicate.

#### C) *In vivo *feeding test

Three different groups of rats were used to study the oral rice tolerance test (ORTT), including Sprague-Dawley (SD) rats, streptozotocin (STZ)-induced diabetic rats and Zucker diabetic fatty (ZDF) rats. Male SD and ZDF rats, aged 5 weeks, were obtained from the Animal Services Centre of the Chinese University of Hong Kong and acclimatized at 23 ± 1°C with a 12 h light/dark cycle. Food and water intake were given *ad libitum*. Male SD rats (350-400 g) were made diabetic by a single intravenous injection of STZ (Sigma, 50 mg/kg body weight). The diabetic status of rats was confirmed by the demonstration of fasting blood glucose level greater than 16.6 mmol/l after 3 days of STZ injection. For ZDF rats, they were maintained on a high-fat 5008 Purina diet (Purina, St Louis, Mo) for 20 weeks. Diabetes was confirmed by a 2-hour oral glucose tolerance test (OGTT), in which blood glucose level attained 11.1 mmol/l. For ORTT, normal SD rats, STZ-induced diabetic rats and ZDF rats were fasted for 16 hours prior to feeding with fine rice grains powder of either transgenic rhIGF-I rice or wild type rice (4.8 g/kg body weight) suspended in water by using oral stainless-steel animal feeding needle. Commercial rhIGF-I was used as control. The concentrations of blood glucose were collected and measured at 0, 0.5, 1, 2, 2.5 and 3 hours using blood glucose strips on ONE TOUCH system (Johnson & Johnson, Milpitas, CA). All procedures were conducted in accordance with the guidelines set by the Animal Services Centre of the Chinese University of Hong Kong.

### Determination of starch content of rice

Total starch contents of both transgenic and wild type rice grains were analyzed according to the manual of the Total Starch Assay Kit based on the use of thermostable amyloglucosidase and α-amylase method (Megazyme). Transgenic rhIGF-I rice and wild type rice grains were milled to fine powder first, followed by incubated with α-amylase and amyloglucosidase. After adding Glucose Determination Reagent to the treated samples, the absorbance at 510 nm for each samples were read against the reagent blank, with D-glucose being the positive control.

### Statistical Analysis

All differences in mean glucose uptake, blood glucose levels and rice starch content were expressed as mean ± SD and were estimated by two-tailed Students *t*-test analysis. A *p *value less than 0.05 was considered to be statistically significant.

## Abbreviations

rhIGF-I: Recombinant human insulin-like growth factor-I; GHIS: growth hormone insensitivity syndrome; ER: endoplasmic reticulum; KDEL: Lys-Asp-Glu-Leu; SP: signal peptide; SD: Sprague-Dawley; STZ: streptozotocin; LRP: lysine-rich protein; BiP: ER luminal binding protein; PDI: protein disulphide isomerase; GLUT4: glucose transporter 4; ORTT: oral rice tolerance test; OGTT: oral glucose tolerance test

## Authors' contributions

SCKC performed the molecular analysis and drafted the manuscript. LZL carried out *in vitro *biological assays. LLL participated in all animal studies. QQL was responsible for rice culturing and collecting rice seeds. SSMS and JCNC conceived of the study, and participated in its design and coordination. PCYT supervised the work. All authors have read and approved the final manuscript.

## Supplementary Material

Additional file 1 **Supplemental Figure S1: Southern blot analysis of genomic DNA from transformants I, SI and SIK**. Genomic DNA extracted from rice leaves of independent transformants was digested with BamHI, blotted on the positively-charged nylon membrane and hybridized with DIG-labeled hIGF-I probe. Lane 1: wild type (wt) rice plant; lanes 2-3: pSB/*Gt1*/hIGF-I transformants I1 and I2; lanes 4-7: pSB/*Gt1*/SP/hIGF-I transformants SI2 to SI5; lanes 8-11: pSB/*Gt1*/SP/hIGF-I::KDEL transformants SIK1 to SIK4. Click here for file

Additional file 2 **Supplemental Figure S2: Comparison of the starch content between the wild type rice (WT) and transgenic IGF-I rice (SI)**. The OD reading are 0.590 ± 0.023 and 0.574 ± 0.019, *p *= 0.119. Data are shown as means ± SD. Click here for file
