# Supplementary figures and images for: Glucose lowering effect of transgenic human insulin-like growth factor-I from rice: in vitro and in vivo studies
Source: BMC Biotechnol. 2011 Apr 12;11:37. doi: 10.1186/1472-6750-11-37 (PMC3098155; doi:10.1186/1472-6750-11-37)

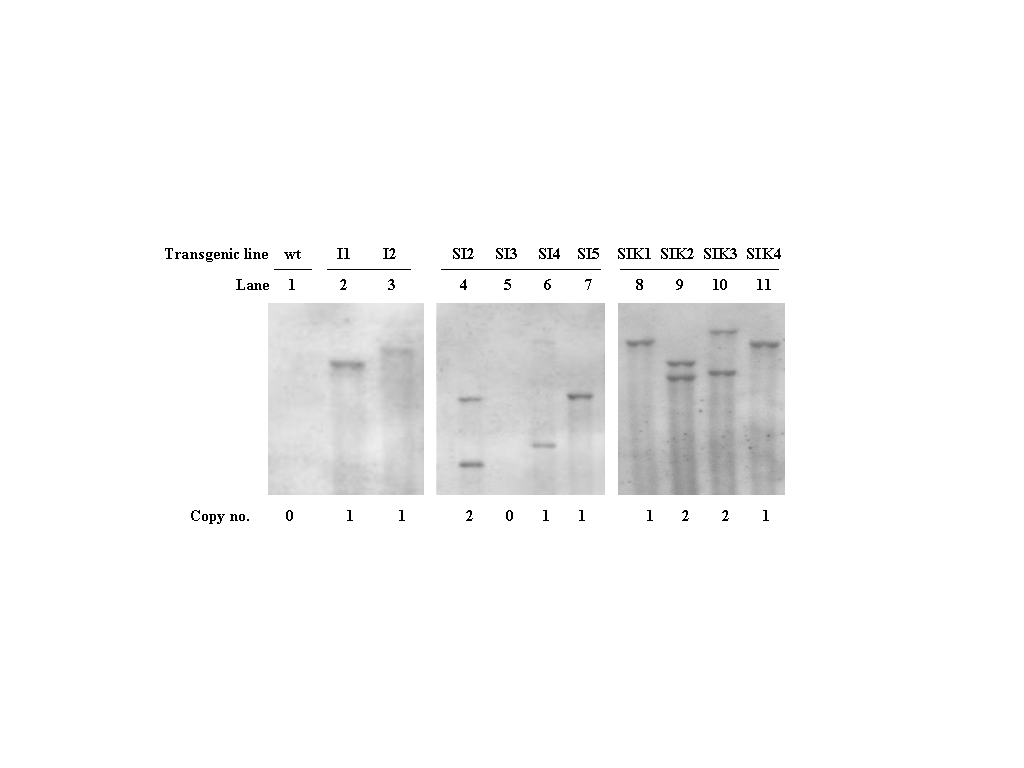

Supplement: Additional file 1 — Supplemental Figure S1: Southern blot analysis of genomic DNA from transformants I, SI and SIK. Genomic DNA extracted from rice leaves of independent transformants was digested with BamHI, blotted on the positively-charged nylon membrane and hybridized with DIG-labeled hIGF-I probe. Lane 1: wild type (wt) rice plant; lanes 2-3: pSB/Gt1/hIGF-I transformants I1 and I2; lanes 4-7: pSB/Gt1/SP/hIGF-I transformants SI2 to SI5; lanes 8-11: pSB/Gt1/SP/hIGF-I::KDEL transformants SIK1 to SIK4. [file 1472-6750-11-37-S1.TIFF]

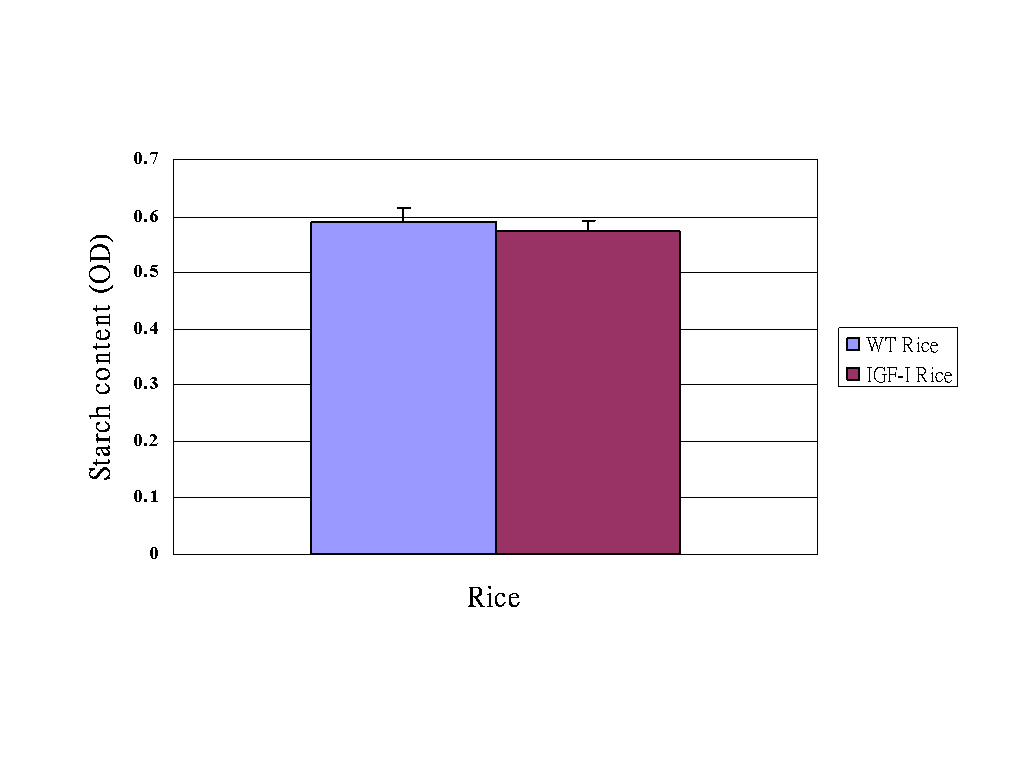

Supplement: Additional file 2 — Supplemental Figure S2: Comparison of the starch content between the wild type rice (WT) and transgenic IGF-I rice (SI). The OD reading are 0.590 ± 0.023 and 0.574 ± 0.019, p = 0.119. Data are shown as means ± SD. [file 1472-6750-11-37-S2.TIFF]
